# Fear of COVID-19 among professional caregivers of the elderly in Central Alentejo, Portugal

**DOI:** 10.1038/s41598-024-52993-6

**Published:** 2024-02-07

**Authors:** Felismina Rosa Mendes, Margarida Sim-Sim, Maria Laurência Gemito, Maria da Luz Barros, Isaura da Conceição Serra, Ana Teresa Caldeira

**Affiliations:** 1 Nursing Department, University of Évora, 7000-811 Évora, Portugal; 2 Comprehensive Health Research Centre (CHCRC), University of Évora, 7000-811 Évora, Portugal; 3 School of Science and Technology, University of Évora, 7000-811 Évora, Portugal; 4 HERCULES Laboratory, University of Évora, 7000-811 Évora, Portugal

**Keywords:** Human behaviour, Comorbidities, Epidemiology, Public health

## Abstract

The coronavirus disease 2019 (COVID-19) has infected many institutionalised elderly people. In Portugal, the level of pandemic fear among professional caregivers of the elderly is unknown, as are its predictive factors. This study aimed to investigate predictors of fear of COVID-19 among workers caring for institutionalised elderly people in nursing homes. This is a cross-sectional study using multiple linear regression applied to a population of 652 caregivers located in 14 municipalities in Central Alentejo, Portugal, at March 2021. The criterion variable was the fear of COVID-19. Standardised regression coefficients showed that the higher the level of education, the lower the level of fear (β = − 0.158; t = − 4.134; p < .001). Other predictors of the level of fear were gender, with women having higher levels (β = 0.123; t = t = 3.203; p < 0.001), higher scores on COVID-19-like suspicious symptoms (β = 0.123; t = 3.219; p < 0.001) and having received a flu vaccine (β = 0.086; t = 2.252; p = 0.025). The model explains 6.7% of the variation in fear of COVID-19 (R^2^Adj = 0.067). Health literacy can minimise the impact on the physical and mental health of these workers. In Central Alentejo, caregivers of the elderly play a fundamental role in social balance. Further studies are needed to better understand the factors that can improve their personal and professional well-being.

## Introduction

The initial lack of knowledge, mode of spread, and the pandemic scale of the coronavirus disease 2019 (COVID-19) generated fear and anxiety, affecting the attitudes and behaviours of the entire population^1^. The heterogeneity of symptoms and outcomes became a factor of uncertainty in risk perception and self-perception of the disease. Given the many cases of COVID-19 in nursing home residents in Portugal and around the world, professional caregivers of older people (PCOP) had a high risk of exposure to SARS-CoV-2.

If elderly people living in nursing homes received considerable media attention^2^, PCOP and their daily work experiences in the context of the pandemic did not. Working in nursing homes requires close contact with residents, and many of these homes lacked personal protective equipment, putting PCOP at high risk of exposure to SARS-CoV-2 and transmission to their families. Some workers described their working conditions in this context as “terrifying”^2^. Prevention of virus transmission became an important daily objective, both within the restricted family circle and in nursing homes. In nursing homes in Portugal, all national standards established by the Directorate-General of Health (DGS) for the prevention of COVID-19 were progressively implemented, and personal protective equipment was progressively provided (standard 007/2020 of 29/3/2020 by DGS, standard 009/2020 of 11/03/2020 by DGS).

The transmission of SARS-CoV-2 in nursing homes was, and still is, a worrying risk not only for the elderly, but also for PCOP. In addition to direct infection, some aspects can make PCOP vulnerable, namely personal factors such as chronic diseases (hypertension, respiratory diseases, obesity, diabetes, kidney disease, liver disease, cerebrovascular disease or cancer)^3^. Behavioural factors such as smoking^3^ or travelling to work, social interaction^4^ lead to an increased risk of infection.

Sociocultural factors and gender-related behaviours also influence exposure to COVID-19. Women are less susceptible because they are more likely to comply with health regulations, wash their hands more often and use more protective equipment^5^. While some studies associate female healthcare workers with higher levels of fear and anxiety in the face of COVID-19^6^, others see such behaviour as preventative, mimicking traditional caregiver roles^5^. Other associations have been observed, namely that higher levels of education are associated with greater knowledge about COVID-19, more preventive measures and less fear^7^. People with chronic illnesses also show more fear and anxiety about COVID-19^8^, as do people with smoking habits^9^.

Fear is an emotion, a subjective, natural, innate, powerful feeling. It is in fact a protective factor against unknown situations and is also a basic biological emotion, an ancestral legacy. It appears in phases, is transient, and appears when one is confronted with a threat^10^. Fear is a key factor in the pandemic crisis, because it is a unique and unexpected phenomenon. Some studies have assessed fear of COVID-19 in the Portuguese adult population^11^. However, to the best of our knowledge, there are no studies that consider fear in PCOP, a group recognised vulnerable to COVID-19, precisely because of their job (Standard 009/2020 of 11/3/2020 by DGS).

The nursing homes in Portugal are a social response designed for collective accommodation. In these homes, elderly people aged 75 and over represented 86% of the total residents^12^. In Portugal, according to the current legislation (Official Gazette) No. 58/2012^13^ the staff of nursing homes includes a variety of professionals. These professionals ensure the provision of services 24 h a day, defining the minimum ratios for each professional area according to the number of elderly residents. During the pandemic crisis, PCOP were trained on the standards and technical guidelines issued by DGS and the respective institutional contingency plans, but their fear levels and related factors are unknown. The aim of this study was to investigate predictors of fear of COVID-19 among PCOP working in nursing homes in Central Alentejo in southern Portugal.

## Methods

### Study design and selection of participants

This was a cross-sectional, exploratory study of a sample of 652 PCOPs from a population of 1020 nursing home workers. PCOPs were recruited based on a series of SARS-CoV-2 tests performed in the workplace. The workplaces were located in 14 municipalities in Central Alentejo. To the best of our knowledge, the topic of fear of COVID-19 has not been studied in this context and given the contingencies of reduced entry into homes for older people, we had to maximize participant selection opportunities and recruit as many workers as possible. Thus, convenience sampling was used to recruit the maximum number of PCOPs without a priori determined sample size.

For data collection, the inclusion criteria of the participants were as follows: (1) adult PCOP aged ≥ 18 years and (2) ability to read and write in Portuguese. There was no criterion related to the length of time the PCOP had worked in the institution, since during the pandemic crisis many institutions received new caregivers from other institutions on a weekly basis (absences due to infection).

Data, which were self-reported by the PCOP, were collected on paper. The questionnaire was hand-delivered to the potential respondent by members of the research team. Once completed, the questionnaire was returned to the researcher in a sealed envelope without any possibility of identification.

The data were collected in March 2021, which corresponded to the end of the third outbreak of the pandemic in Portugal, which began at the end of December 2020 with the Christmas celebrations. For the purposes of the current study, the population density of the 14 municipalities was dichotomised, considering those with less than 25 inhabitants/km^2^^14^ as low density.

A total of 682 questionnaires were collected from the PCOPs that were manifestly available, of which 30 were excluded due to a lack of response to approximately 30–50% of the questions. The response rate was 62.6% (Table 1).

**Table 1:** Representation of municipalities according to population density, cumulative incidence of COVID-19 (1 and 30 March 2021), PCOP staff, returned questionnaires, and response rate.

Municipalities	Inhab/km^2^^13,14^	Cumulative incidence March/2021 DGS report 364	Cumulative incidence 30 March 2021^15^	Workers at the institution (n)	Questionnaires applied	Answered questionnaires (n, %)	% of employees represented
Alandroal	9.3	120–239.9	200.3	108	70	67 (95.7)	62.0
Arraiolos	9.8	480–959.9	43.3	43	26	20 (76.9)	46.5
Borba	44.5	60–119.9	133.6	96	79	79 (100.0)	82.3
Estremoz	25	240–479.9	23.6	72	46	45 (97.8)	62.5
Évora	41.2	120–239.9	5.7	118	81	81 (100.0)	68.6
Montemor	13	60–119.9	12.8	88	47	46 (97.9)	52.3
Mora	9.4	20–59	0.0	86	57	56 (98.2)	65.1
Mourão	8.6	20–59	40.8	44	32	32 (100.0)	72.7
Portel	9.6	20–59	34.3	96	71	69 (97.2)	71.9
Redondo	17	60–119.9	31.5	43	37	36 (97.3)	83.7
Reguengos	21.3	120–239.9	99.9	50	31	31 (100.0)	62.0
Vendas Novas	51.1	120–239.9	17.8	37	26	24 (92.3)	64.9
Viana	13.8	240–479.9	38.9	110	45	38 (84.4)	34.5
Vila Viçosa	37.9	120–239.9	0.0	51	34	28 (82.4)	54.9
Total	–	–	–	1042	682	652 (95.5)	62.6

At the time of data collection, the municipality of Reguengos was in code yellow (≥ 60–120 cases/100^3^ inhabitants). The municipalities of Alandroal and Borba were in code orange (≥ 120–240 cases/100^3^ inhabitants). All other municipalities in Central Alentejo were in code green (< 60 cases/100^3^ inhabitants).

### Instrument

The data collection instrument was divided into seven sections: (1) sociodemographic characteristics; (2) variables related to the occurrence of infections and the occurrence of symptoms similar to COVID-19; (3) variables related to environmental risk exposure; (4) variables related to the presence of current chronic diseases and addictive behaviours; (5) variables related to preventive behaviours in relation to COVID-19; (6) knowledge of COVID-19 scale^15^ and (7) the Fear of COVID-19 scale (FCV-19S)^11^.

### Sociodemographic variables

Sociodemographic variables included (1) age (continuous variable in years); (2) gender (male, female); (3) educational level (primary, 6th, 9th, 12th grade, and higher education).

### Variables related to the incidence of infections and symptoms similar to COVID-19

The following variables related to the incidence of COVID-19 were considered: (1) frequency of testing (polymerase chain reaction-PCR) and (2) occurrence of SARS-CoV-2 infection. The variables related to screening for current symptoms similar to COVID-19 were answered on a yes-or-no basis and included 17 more common symptoms in Portugal as DGS-Report 029^16^, and literature^17^: (1) fever, (2) cough, (3) shortness of breath, (4) headache, (5) body aches, (6) fatigue, (7) change in taste, (8) loss of smell, (9) stuffy nose, (10) hoarseness, (11) painful swallowing, (12) sore tongue, (13) diarrhoea, (14) vomiting, (15) itchy palms, (16) itchy body, and (17) chills.

### Variables related to environmental risk exposure

This section considered (1) occurrence of contact with persons in the contagious phase (category 0 = no contact/do not know and category 1 = yes); (2) contact of the respondent’s relatives with persons in the contagious phase (category 0 = no contact/do not know, category 1 = yes); (3) cohabitation with infected persons (category 0 = no, category 1 = yes); and (4) mode of transport to and from work (category 1 = own car, alone; category 2 = shared car or public transport; and category 3 = soft modes). The incidence of cases per 100^3^ inhabitants at the time of questionnaire completion was also considered like DGS-Report 385^16^ (category 0 = incidence < 59.9 cases/100^3^ inhabitants, category 1 = incidence between 60–119.9 cases/100^3^ inhabitants, category 2 = incidence between ≥ 120–239.9 cases/100^3^ inhabitants, and category 4 = incidence ≥ 240 cases/100^3^ inhabitants).

### Variables related to current chronic diseases and addictive behaviours

Health status was assessed on a “0 = no” and “1 = yes” basis, covering eight chronic diseases: respiratory disease, heart disease, diabetes, cancer, hypertension, kidney disease, autoimmune disease, and obesity. From the data collected, a variable was created that represented the sum of the participants’ self-reported chronic diseases, ranging between 0 and 8. Smoking was assessed as a categorical variable (0 = no and 1 = yes).

### Variables related to preventive behaviour

Two doses of COVID-19 vaccine were considered as COVID-19 vaccination (0 = no, 1 = yes). The time (in days) between the first vaccine dose and the date of completion of the questionnaire and the time between the second vaccine dose and the date of completion of the questionnaire date were recorded as continuous variables. Participants were also asked whether they had received the flu vaccine in the previous flu season (no = 0, yes = 1).

### Knowledge of COVID-19

Participants’ knowledge of COVID-19 was assessed using a scale applied to the Portuguese adult population^15^. As the scale was not given an acronym in the original study, we refer to it as “ECC-19” in the present study. ECC-19 consists of 14 items of the type "Wearing a face mask does not help prevent COVID-19", with response options "True", "False", and "don’t know". Correct answers are assigned a score of 1, and incorrect answers and "I don’t know" are assigned a score of 0. Higher scores indicate greater knowledge. Internal consistency was not reported in the original study^15^. The present study found an internal consistency of 0.681. To protect intellectual property rights, permission to use the scale was requested from the author and granted on 7 February 2021.

### Fear of COVID-19 scale

The Fear of COVID-19 Scale (FCV-19S) assesses the respondent’s emotional response to infection. The FCV-19S is a latent variable with seven manifest variables or items presented on a Likert scale ranging from 1 (strongly disagree) to 5 (strongly agree). All items have positive wording, e.g., “I am afraid of dying from COVID-19”. The total score is obtained by summing the item scores and can vary between 7 and 35 points. A higher score means greater fear. The Portuguese version had an internal consistency of 0.800^11^. In the present study, the internal consistency had a Cronbach's alpha of 0.870. Permission to use the instrument was requested by e-mail from the author, and a positive response was received on 7 February 2021.

As 29 questionnaires (4.4%) had at least one item of FCV-19S unanswered (each item representing 14.3%), the author’s^18^ criterion was used, i.e., the mean score given by the participant to the other items was assigned to the blank responses. The mean of the answered items can be used to replace the missing data if the blank responses do not exceed 20% as supported by literature^18,19^.

### Statistical analysis

Descriptive statistics with measures of central tendency and dispersion were used for the continuous quantitative variables. Absolute frequencies and proportions were used to categorical variables. Given the non-normal distribution of the quantitative variables (Shapiro–Wilk test), non-parametric tests (Mann–Whitney U test) were used to assess the relationships between the FCV-19S score and the categorical variables. Non-parametric Spearman correlation was used to quantify the relationships between the FCV-19S score and continuous variables.

To identify the predictors of fear of COVID-19, we considered FCV-19S as a criterion variable, and according to the results of the bivariate analysis, potential explanatory factors were those with p ≤ 0.25^20^. The stepwise approach was used due to the exploratory nature of the study, as we lacked a consistent theory regarding the relationship between the variables. Thus, the selection of predictor variables resulted from the statistical procedure^20,21^. A regression coefficient was calculated for each predictor to determine how each predictor affected the outcome holding the others constant. A 95% confidence level was used for a 5% significance level. A *p* value of < 0.05 was considered statistically significant.

As the sample size had been determined in advance, by collecting as many questionnaires as possible, the observed power, or test power, hasn’t declared a priori. Although there is controversy about the significance and usefulness of test power in *post-hoc* analyses^22^, it was considered appropriate to include this information. Therefore, with the support of G*Power^23^, the size of the sample was considered (n = 652) and the effect size calculated (f^2^ = 0.072), observing that the power of the post hoc test was 0.999. The power is high (> 0.80)^20^.

Data were analysed using version 27 of the IBM^®^ SPSS statistical package^24^.

### Ethical considerations

Data were collected using a paper questionnaire, after potential participants were asked to sign an informed consent form, in duplicate, with one copy kept by the respondent and the other by the researcher. The first page of the data collection instrument explained the voluntary nature of participation, and anonymity was guaranteed.

The present study, as a part of a larger research, was conducted in accordance with the tenets of the Declaration of Helsinki. All methods were performed according to the relevant guidelines and regulations.

Approval was obtained from the Ethics Committee for Scientific Research in the Areas of Human Health and Welfare of the University of Évora (Opinion 21021), on 17 March 2021, prior to the start of the study. Consent for data collection was obtained from all institutions. The study did not provide economic compensation to the participants.

## Results

### Sociodemographic characteristics of the participants

Participants had a mean age of 44.75 (SD = 11.55) years, ranging from 19 to 68 years. The vast majority were female (n = 590; 90.5% *versus* n = 62; 9.6% male) and Portuguese (n = 640; 98.2% *versus* n = 12; 1.8% other nationalities). The predominant level of education was 12th grade (n = 198; 30.4%), followed by 9th grade (n = 187; 28.7%), higher education (n = 100; 15.4%), 6th grade (n = 99; 15.2%) and primary education (n = 68; 10.4%).

### Participants’ symptoms similar to COVID-19 infection, chronic diseases and health behaviours

At the time of data collection, most workers (n = 519; 79.6%) had not been infected with COVID-19. In the univariate analysis, the following characteristics of the participants were observed, like symptoms similar to COVID-19 infection in the previous two weeks and identification of contacts at risk of infection (Table 2).

**Table 2:** Participants' symptoms and COVID-19 infection risk contacts.

Variable group	Variable	Category	n	%
Occurrence of symptoms similar to SARS-CoV-2 infection suspicion (n = 652)	Tongue inflammation	Yes	20	3.1
	Vomiting	Yes	36	5.5
	Itchy palms	Yes	38	5.8
	Itchy body	Yes	44	6.7
	Shortness of breath	Yes	48	7.4
	Fever	Yes	54	8.3
	Hoarseness	Yes	60	9.2
	Altered taste	Yes	69	10.6
	Loss of smell	Yes	73	11.2
	Diarrhoea	Yes	79	12.1
	Chills	Yes	106	16.3
	Cough	Yes	123	18.9
	Stuffy nose	Yes	128	19.6
	Painful swallowing	Yes	141	21.6
	Fatigue	Yes	176	27
	Body ache	Yes	214	32.8
	Headache	Yes	262	40.2
Exposure to risk in the environment	Home–work commute (n = 643)	Own car always alone	413	64.2
		Public transportation or carpooling	42	6.5
		Soft modes	188	29.2
	Contact with individuals with COVID-19 in the contagion phase (n = 652)	Yes	285	43.7
		Does not know/was not	367	56.3
	Contact with relative who met an individual infected with COVID-19 (n = 652)	Yes	182	27.9
		Does not know/was not	470	72.1
	Cohabitation with individuals infected with COVID-19 (n = 652)	Yes	118	18.1
	Weekly incidence (n = 652)	< 120 cases/100^3^ inhab	506	77.6
		≥ 120–240 cases/100^3^ inhab	146	22.4

In the univariate analysis, other characteristics were also observed, such as (1) identification of current chronic disease, (2) smoking, (3) preventive risk behaviour (Table 3).

**Table 3:** Participants' chronic diseases, addictive, and preventive behaviour.

Variable group	Variable	Category	n	%
Chronic diseases	Cancer	Yes	11	1.7
	Kidney	Yes	17	2.6
	Autoimmune	Yes	28	4.3
	Heart	Yes	30	4.6
	Diabetes	Yes	30	4.6
	Respiratory	Yes	47	7.2
	Obesity	Yes	54	8.3
	Hypertension	Yes	122	18.7
Addictive behaviours	Smoking	Yes	172	26.4
Preventive behaviours	Covid-19 Vaccine 1st dose	Yes	501	76.8
	Covid-19 Vaccine 2nd dose*	Yes	417	64
	Flu vaccine	Yes	351	53.8

*Many workers became infected after the first dose and the time had not yet passed for them to be eligible for the second dose of the vaccine, at the time the questionnaire was administered.

### Variables related to knowledge of COVID-19

Participants' knowledge of COVID-19, assessed using a scale^15^ showed an average of 11.27 (SD = 1.54), ranging from 0 to 14 points, with a mode and median of 12.

### Variables related to fear of COVID-19

Fear, assessed using FCV-19S, had a mean of 20.56 (SD = 5.94), ranging from 7 to 35 points, with a mode and median of 21. The bivariate analysis assessed the relationship between FCV-19S and the variables analysed in the previous section. Once the variables were dichotomised, the Mann–Whitney test was performed, showing that the mean ranks were significantly higher (a) in women (p < 0.001), (b) in participants with only basic education (p < 0.001), (c) in participants who had had COVID-19 (p = 0.009), and (d) in those who were immunized against the flu (p = 0.016) (Table 4).

**Table 4:** Study population answers regarding COVID-19 fear level considering categorial variables.

Variable		Category	n (%)	Mean rank	Coefficient	p value
Demographic characteristics	Gender (n = 652)	Male	62 (9.5)	246.79	< 0.001	< 0.001
		Female	590 (90.5)	334.88		
	Level of education (n = 652)	≤ Basic education	552 (84.6)	340.32	U = 19,970.5; Z = − 4.408	< 0.001
		Higher education	100 (15.4)	250.21		
Screening and diagnosis	Previous COVID-19 infection (n = 652)	No	519 (79.6)	316.78	U = 39,559.5; Z = 2.607	0.009
		Yes	133 (20.4)	364.40		
Addictive behaviours	Smoking (n = 652)	No	480 (73.6)	333.36	U = 37,987.5; Z = − 1.555	0.120
		Yes	172(23.4)	307.36		
Risk of infection	Commute home–work (n = 644)	Own car	414 (64.2)	312.58	U = 51,725.0; Z = 1.821	0.069
		Shared transportation/walking	230 (35.8)	340.39		
Risks in the environment	Cohabitation with infected individuals (n = 652)	No	534 (81.5)	322.78	U = 33,490.5; Z = 1.073	0.283
		Yes	118 (18.5)	343.32		
	Contact with infected individuals (n = 652)	No/Does not know	367 (58.72)	333.02	U = 49,904.0; Z = − 1.005	0.315
		Yes	285 (43.7)	318.10		
	Contact with relative who contacted infected individuals (n = 652)	No/Does not know	470	331.37	U = 40,481.0; Z = − 1.062	0.288
		Yes	182	313.92		
	Weekly incidence (n = 652)	< 120 cases/100^3^ inhab	506 (77.6)	330.83	U = 34,747.5; Z = − 1.094	0.274
		≥ 120 cases/100^3^ inhab	146 (22.4)	311.5		
	Population density (n = 652)	< 25 Inhab/Km^2^	395 (60.6)	331.05	U = 48,959.5; Z = − 0.766	0.444
		≥ 25 Inhab/Km^2^	257 (39.4)	319.5		
Preventive behaviours	Received 2 COVID-19 vaccine doses (n = 652)	No	235 (36.0)	328.74	U = 48,470.5; Z = − 0.229	0.819
		Yes	417 (63.9)	325.24		
	Received the flu vaccine (n = 652)	No	301 (45.3)	307.27	U = 58,613.5; Z = 2.417	0.016
		Yes	351 (54.7)	342.99		

The relationship between FCV-19S and numerical variables was also assessed. Spearman correlation showed that there were no significant associations between FCV-19S and the continuous variables, except for the association with the Suspicious Symptoms variable (rs = 0.117; n = 652; p = 0.003). However, in the correlation, the p-value is lower than 0.25 in the relationship between FCV-19S and the Days Post-Vaccine1 variable (p = 0.246) (Table 5).

**Table 5:** Respondents’ answers about COVID-19 fear level: numeric variables.

			Age	Days post-Vaccine 1	Knowledge about Covid-19	Chronic diseases	Suspicious symptoms
Spearman's rho	FCV19S	Correlation coefficient	0.034	0.046	− 0.017	0.011	0.117**
		Sig. (2-tailed)	0.394	0.246	0.667	0.777	0.003
		N	635	652	652	652	652
	Age	Correlation coefficient	1	0.017	− 0.025	0.258**	− 0.075
		Sig. (2-tailed)		0.665	0.525	< 0.001	0.058
		N		635	635	635	635
	Days post-vaccine 1	Correlation coefficient		1	0.047	− .096*	− 0.197**
		Sig. (2-tailed)			0.233	0.015	< 0.001
		N			652	652	652
	Knowledge about Covid-19	Correlation coefficient			1	− 0.107**	− 0.031
		Sig. (2-tailed)				0.006	0.426
		N				652	652
	Chronic diseases	Correlation coefficient				1	0.189**
		Sig. (2-tailed)					< 0.001
		N					652

**Correlation is significant at the 0.01 level (2-tailed).

*Correlation is significant at the 0.05 level (2-tailed).

### Regression analyses

To identify the predictors of FCV-19S, a stepwise multiple linear regression was performed. The multiple linear regression model identified the predictors of fear (F_(4,643)_ = 12.492; p < 0.001). The variables that were significant in the Mann–Whitney test and Spearman correlation were included in the model, as were the variables with p ≤ 0.25^20^.

Upon examining the standardized coefficients, education level emerged as the most influential predictor (β = − 0.158; t = -4.134; p < 0.001). Additional significant predictors included gender (β = 0.123; t = 3.203; p < 0.001), the tally of suspected COVID-19 symptoms (β = 0.123; t = 3.219; p < 0.001), and flu vaccination status (β = 0.086; t = 2.252; p = 0.025). The derived regression equation is as follows:

$${\text{Y}} = {\text{b}}0 + {\text{b1}}*{\text{x1}} + {\text{b2}}*{\text{x2}} + {\text{b3}}*{\text{x3}} + {\text{b4}}*{\text{x4}}$$

With the data obtained from the table of coefficients, the equation has the following expression:

$${\text{FCV}} - {\text{19S }} = { 17}.{545} + \left( { - {2}.{6}00*\left( {\text{education level}} \right)} \right) \, + { 2}.{553}*\left( {{\text{gender}}} \right) \, + \, .{219}*\left( {\text{suspicious symptoms}} \right) \, + { 1}.0{27}*\left( {\text{received Flu Vaccine}} \right)$$

When holding all other variables constant in the model and considering the non-standardized coefficients, the following interpretation can be drawn: (a) Education level was significantly associated with fear among PCOPs: those with lower educational attainment experienced fear at a rate 2.600 times higher than their counterparts with more education; (b) Gender appeared to be a significant predictor of fear, with women experiencing a 2.553-fold increase in fear compared to men; (c) The presence of symptoms associated with COVID-19 also influenced fear levels, with each additional symptom reported by PCOP increasing the FCV-19S score by 0.219; d) Additionally, PCOP who had received the flu vaccine reported a 1.027-fold increase in fear of COVID-19 compared to those who had not been vaccinated. Detailed results from the regression analysis are displayed in Table 6, and supplementary Table 1 (SupT1) contains the complete dataset.

**Table 6:** Multivariable linear regression analysis with fear against COVID-19 as the dependent variable.

Coefficients^a^										
Model 4		Unstandardised Coefficients		Standardised Coefficients	t	Sig	95.0% Confidence Interval for B		Collinearity Statistics	
		B	Std. Error	Beta			Lower Bound	Upper Bound	Tolerance	VIF
(Constant)		17.545	0.794		22.099	< 0.001	15.986	19.104		
Educational (0 ≤ basic; 1 = Higher Education)		− 2.600	0.629	− 0.158	− 4.134	< 0.001	− 3.835	− 1.365	0.994	1.006
Gender (0 = male; 1 = female)		2.553	0.797	0.123	3.203	0.001	0.988	4.118	0.982	1.018
Suspicious symptoms (0–16)		0.219	0.068	0.123	3.219	0.001	0.085	0.353	0.988	1.013
Flu Vaccine (0 = no; 1 = yes)		1.027	0.456	0.086	2.252	0.025	0.131	1.922	0.989	1.011

^a^Dependent Variable: FCV19S.

The following variables were excluded from the model: (1) previous infection with COVID-19 (p = 0.530), (2) smoking (p = 0.083), (3) commuting home-work (p = 0.220) and (4) number of days since the first dose of the COVID-19 vaccine (p = 0.409).

**Figure 1:**
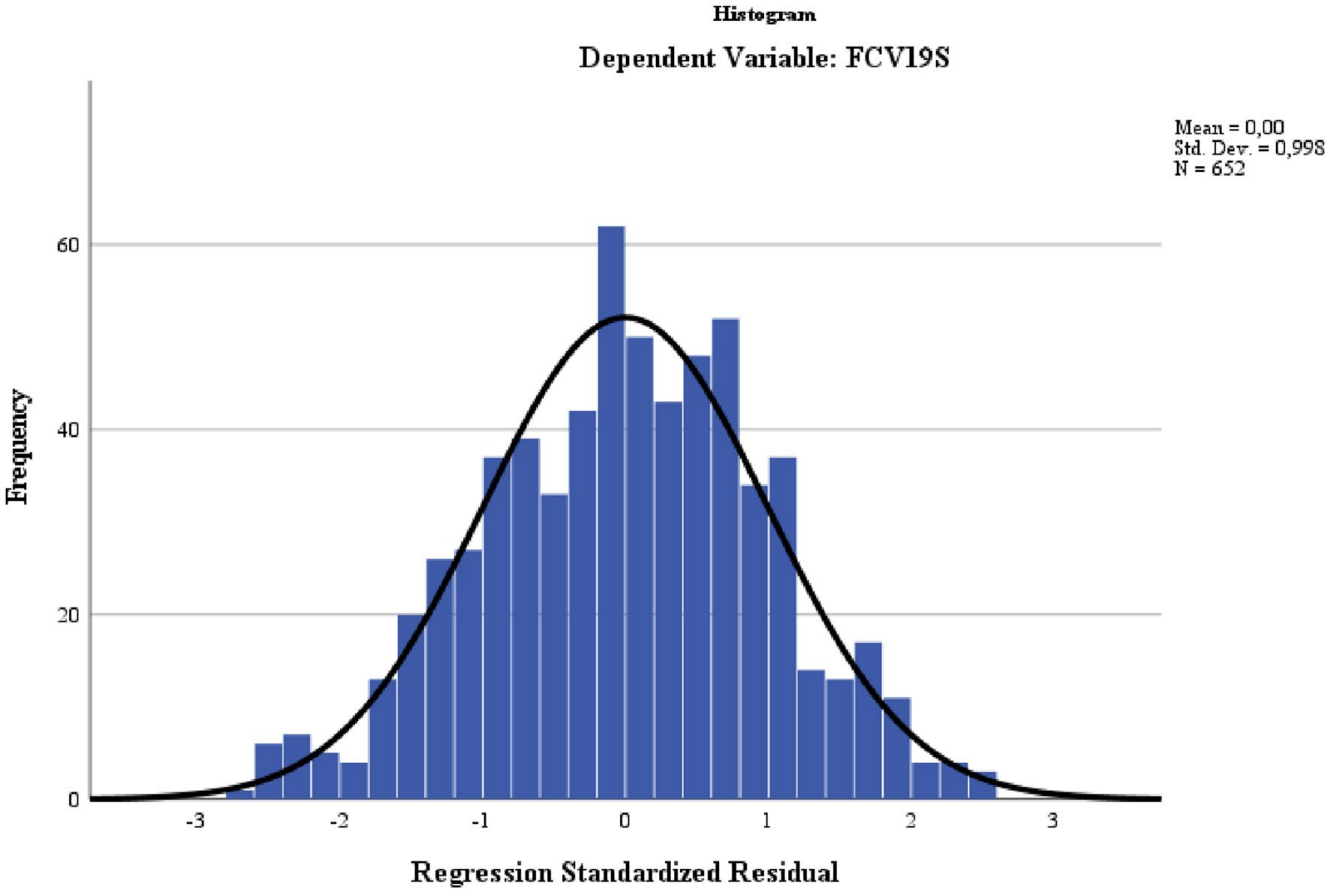
Histogram of the residuals.

Regarding the assumptions^20^, the model showed an absence of multicollinearity, with a variance inflation factor < 10, ranging from 1.006 to 1.018. In contrast, the tolerance was > 0.20, ranging between 0.982 and 0.994. No outliers (standardised residuals beyond 3 standard deviations) were identified (Std residuals from − 2.630 to 2.514). The Durbin-Watson statistic showed that the residuals (difference between the predicted and observed values) were independent, ranging between 1.5 and 2.5 (Durbin-Watson = 1.912). The fourth model significantly improved the prediction of the FCV-19S score (F_(4,639)_ = 12.492, p < 0.001). The plot of the standardised residuals (ZRESID) and the standardised predicted values (ZPRED) showed a random set of scattered points, which confirmed the data linearity and homoscedasticity. The histogram of the residuals suggested that the data approximated a normal distribution (Fig. 1).

The model explains 6.7% of the variation in fear of COVID-19 (R2 = 0.067). The magnitude of the effect, using Cohen's f^2^ (f^2^ = R^2^/(1-R^2^)), revealed a small effect size (f^2^ = 0.0071)^20^.

## Discussion

The fear of COVID-19 infection among caregivers of institutionalized elderly people, a vulnerable group, was addressed, as highlighted by the WHO. Constraints on access to nursing homes implied a strategic approach to participant recruitment. Consequently, we utilized convenience sampling to encompass as broad a representation of PCOPs as was practicable under the circumstances. Although this methodology precludes the generalization of our results, we should underscore the value of nonrandomized studies that include as many accessible cases as possible, as they provide a crucial broad-based perspective of the phenomenon in the case of events affecting public opinion^25^.

Considering the sociodemographic characterization, it is evident that the majority of PCOP were female (with binary sex orientation considered), a finding that is consistent with previous studies^26,27^. In long-term care, the significant representation of female professionals follows the traditional domestic roles. Possibly, it emulates the family, reinforcing the gender-associated traits of the role of caregiver, with women spending more time on these types of tasks. Male individuals are mainly assigned decision-making roles, reproducing greater power in family negotiations, which is associated with less time spent caring for significant others^28^. The greater representation of participants with educational qualifications of 9th and 12th grade not only reflects the PCOP career path (Decree-Law No. 414/99, of October 15), but also the need to temporarily recruit direct action staff, in order to meet the allocation legally defined in Article 12 of Ordinance No. 67/2012 of March 21, by the Ministry of Solidarity and Social Security (Diário da República, 1st series—No. 58—March 21, 2012)^13^. The high representation of these professionals in the current study (84.7%) is in line with other studies^29^ and also with the facilities that Ordinance 82-C/2020, of March 31 granted, through an exceptional regime, for the recruitment of staff during the pandemic. This was necessary both due to the increased needs of the elderly patients and the temporary inability of professionals to work due to COVID-19 infection, prophylactic isolation, or family illness situations. In fact, it was even necessary to consider more places for the elderly in residential institutions, as there were cases of hospital discharges that required accommodation^30^ and thus, a greater need for PCOP.

The representation of about 1/5 of the employees (20%) who report a previous infection with COVID-19 suggests a higher contagion rate, which occurred after the 2020 Christmas holidays, marking the beginning of the third wave in Portugal, to which a new variant of the virus was added^31^. Although in the current study, it represents a lower percentage than the average of 30% of cases observed in professionals working in elderly residences^30^ it is a significant number. Indeed, as of December 31, 2020, there were 410,245 infected individuals and 6,871 deaths (DGS-Report 304) increasing to 809,053 and 16,751, respectively, on March 31, 2021 (DGS-Report 394)^16^. The critical period in Central Alentejo was around mid-January 2021 (DGS-Report 322)^16^ with the region showing a 14-day cumulative incidence rate (rate = 1663.2) higher than the national average (rate = 1266.3).

Regarding symptoms similar to COVID-19, the importance of their observation in the current study is explained by the fact that some people infected with SARS-CoV-2 have mild symptoms or are asymptomatic^32,33^. The six most representative symptoms are headache, body ache^32,34^, fatigue^32^ painful swallowing^33^, stuffy nose, and cough^32–34^, which are common complaints in individuals with and without COVID-19. However, the first three symptoms and fever can be considered warning signs given their specificity of over 90%, increasing the likelihood of the presence of COVID-19^33^. Nevertheless, the symptom patterns are very varied; for example, headaches in COVID-19 patients can persist for six months^35^. On the other hand, fatigue, perhaps at the time of data collection, reflected the weariness of the PCOPs after a year of pandemic, 13 states of emergency, one of calamity, and three pandemic waves^31,36^. It is believed that the recording of symptoms in the current study was important, as it contributed to the selection of participants for further follow-up^33^.

In the sample of participants, family contacts, cohabitation with infected subjects, or sharing transportation suggest that some PCOPs could be at risk for COVID-19. This corresponds to some studies, where a greater chance of infection was observed in employees who reported contact with infected family members or suspects not yet confirmed for COVID-19^34^. Regarding means of transport, it is observed that a large part of the PCOPs would have a lower risk of contagion, since they used their own, unshared transport and soft modes. Indeed, the perception of low risk of contagion is found in people who use their own car or soft modes (bicycle, scooter, motorcycle, walking)^37^.

A higher risk was also found in the social interactions of PCOPs living in the municipalities of Alandroal and Borba at the time of data collection. However, the risk was antecedent for the PCOPs living in the various municipalities, as in an earlier phase the situation in Central Alentejo was serious. Already at the beginning of March 2021, the DGS reported cumulative incidence in the range of 480–959.9 in the municipality (Arraiolos), as well as 240–479.9 in Estremoz and Viana do Alentejo, with lower incidence in Évora, Reguengos, Vendas Novas, and Vila Viçosa in the range of 130–239.9 (DGS-Report 364 https://covid19.min-saude.pt/wp-content/uploads/2022/03/364_DGS_boletim_20210301_pdf-471kb.pdf). The evolution of the pandemic in Central Alentejo initially had protective factors. In the first COVID-19 wave (March 2020), cases were scarce in the region, occurring a diffuse expression of the disease, argued with the low population density, locally centred mobility, geographic isolation with concentrated settlement, and the establishment of consecutive states of emergency^38,39^. On the other hand, in the third wave (peak on January 28, 2021), the temporary return to family roots during the Christmas period, with greater social interaction, led to a very high number of cases in Central Alentejo (DGS-Report 322 https://covid19.min-saude.pt/wp-content/uploads/2022/03/322_DGS_boletim_20210118_pdf-452kb.pdf, with the effects still visible up to the date of data collection (DGS-Report 394 https://covid19.min-saude.pt/wp-content/uploads/2022/03/394_DGS_boletim_20210331_pdf-383kb.pdf).

In the current study, the percentage of PCOP vaccinated against COVID-19 (76.8% with the 1st dose; 64% with the 2nd dose) reflects the progressive effort towards immunity, after a somewhat slow start in December 2020^31^. In the pandemic crisis, Portugal was successful, achieving the highest rate in the OECD, with more than 85% of the population receiving the 1st dose by September 2021^31^. We must also consider the representation of the flu vaccine among PCOP. These professionals were vaccinated in September 2020, in an early campaign. According to DGS guidelines (Standard No. 016/2020 of 25/09/2020), PCOP as a risk group, had free vaccination available. Although adherence to vaccination among PCOPs in the current study was not high (n = 351, 53.8%), it reflects the health sector's prevention policies and a low level of self-care. This is in line with other studies that also identify Vaccine Hesitancy, which is expressed in the refusal or delay in acceptance, even when it is free and accessible^40^.

The profile of chronic diseases presented by PCOPs is consistent with common pathologies in Southern Portugal. The condition of hypertension, self-recognized by 122 PCOPs (18.7%), is close to the results of the previous study, in which the representation of participants reached 18.3% in the Algarve and 23% in the Alentejo^41^. Obesity is reported by PCOPs (n = 54, 8.3%), a lower representation than the 17% self-reported by portuguese adults^42^. Perhaps, excess weight in PCOPs has lost its significance for them, since they live in a region where the prevalence of obesity is the highest in the country (Alentejo: 28.2% of the population) and 35.2% of residents are overweight^43^. The high incidence of overweight in the social environment where the PCOPs live can devalue this health condition and not understand it as pathological or pre-pathological. In fact, PCOPs are at increased risk if they have chronic diseases. In Portugal, in people who have died from COVID-19, prevalence rates ≥ 10% have been found in cases with a previous history of hypertension, diabetes, and heart, kidney, or cerebrovascular disease^44^.

In the current study, it was found that PCOPs exhibit levels of fear significantly above the average of 17.20 (SD = 5.69) identified in the common Portuguese population^11^ and 14.2 (SD = 6.14) in French health professionals^26^. Fear feeds on ignorance and misinformation, leading to delays in healthcare, creating dilemmas and hesitations based on rhetoric vehemently defended but incorrect in scientific evidence. If myths related to the transmission of the disease through food^45^, mosquito bites, the drinking water network, among others^46^ emerged during the first pandemic wave, around the time the data was collected, the rumour of the approval of the vaccine against SARS-Cov-2 was circulating in the country on social media, before the compliance with the inherent protocol^47^.

### Predictors of FCV-19S

Given the entire personal and social context that generated insecurity during the pandemic, we will now discuss the association of the **FCV-19S** response variable with a set of variables. Methodologically, the sample size assumption was verified, as the number of cases was more than 10, 15, or 20 for each predictor variable, or greater than a minimum of 175^20,21^. The choice to include the unstandardized coefficients in the results section was due to the greater ease of interpretation of the relationship between the independent variables X and the outcome variable Y^20^.

### Gender as a predictor of FCV19S

With regard to the female gender as a predictor of higher levels of fear, the results are consistent with other studies in china, Saudi Arabia and Portugal^11,48,49^. A gender effect emerges in relation to the level of FCV19S. On the other hand, women also more often play socio-familial roles as caregivers for their own families, both with younger generations (their children) and older generations (their parents). The high level of FCV19S could be rooted in a greater perception of risk in transmitting it to people in their family circle. In fact, it was women who showed the greatest fear, both due to their own perception of risk and family transmission^27^.

### The level of education as a predictor of FCV19S

The association between higher FCV19S levels and lower educational attainment is present in the general population and also in less differentiated employees in health institutions^48,50^. It is also the employees with the least academic training who have the highest infection rates^34^. Institutions are responsible for promoting health literacy among their staff by disseminating reliable information to reduce misinformation and myths and to make gains in the care provided. Literacy of PCOPs brings personal benefits and adds value to institutional care. It thus becomes a community protection factor, increasing health potential.

### Flu vaccination as a predictor of FCV19S

People who are more concerned with or more afraid of the disease may have resorted to flu vaccination to prevent COVID-19, or at least in the expectation that if they become infected, they will develop less severe forms of the disease. In fact, the people who are most afraid and consider themselves at high risk for COVID-19 are those who express their intention to receive the flu vaccine^51^. However, in other people, the feeling of invincibility sometimes sets in, which leads to self-exclusion from risk. These are people who overestimate their body's capabilities, discredit the guidelines of health institutions, and have a low perception of susceptibility. This concurs with other studies, where subjects who did not want to be vaccinated also doubted the safety conferred by vaccination, considering it superfluous for their person^52^.

### Suspicious symptoms similar to COVID-19 as a predictor of FCV19S

For PCOPs who report symptoms that could be indicative of COVID-19, their fear may be compounded by the uncertainty of their health status and the discomfort of the symptoms. Fear among PCOP may be influenced by the images and ambiguity of certain news reports broadcasted by the media about severe COVID-19 cases, as well as the official daily counts of intensive care unit hospitalizations. On one hand, media coverage transmitted the severity of the pandemic and provided information on symptoms and the need for protection. On the other hand, some platforms perpetuated a state of persistent alarmism, creating emotional distress with a negative impact on mental health. An incorrect perception of risk can lead to exaggerated and disruptive responses of fear, escalating to what is known as coronaphobia^53^. This is recognized as a specific phobia in the DSM-5, an anxiety disorder characterized by persistent and excessive fear, where individuals dramatically and distressingly misinterpret common symptoms of benign diseases^1^. The absence of these symptoms in some COVID-19 cases, however, complicates the diagnosis. Consequently, healthcare-seeking behaviour is influenced by public perceptions of the disease, beliefs about symptoms, and the perceived severity or virulence^17^. It is not surprising that the PCOPs in the current study, regularly confronting various contagion factors, experienced fear upon exhibiting suspicious symptoms that resembled those of COVID-19.

## Conclusion

The level of COVID-19 infection fear among PCOP is higher in female professionals. It is influenced by education and intrinsic factors such as gender or symptoms that suspiciously resemble COVID-19. In any situation, fear is an emotional response to a threat, as COVID-19 continues to be. Despite measures implemented to minimize the impact of the disease, increased critical awareness, associated with higher levels of literacy, may potentially lower the fears exhibited by PCOPs. Mitigating fear is vital for ensuring the safety of those who are exposed daily to the risk of contagion, thereby reducing the impacts of the pandemic.

### Limitations and strengths

The convenience sample prevents generalisation of the results. In methodological terms, the assumptions of the multiple linear regression were met.

The post-hoc sample size analysis, especially the observed power verified afterwards, reflects limitations in a pandemic context. Indeed, given the unpredictability of PCOP attendance at work, we chose to apply as many questionnaires as possible in a convenience sample, without a priori determining the effect size and observed power. This limitation led to another weakness from an ethical standpoint, as it wasted the efforts of the PCOPs in responding and the researchers' time due to the volume of data entry.

The study addresses an emotion, which is why the cross-sectional approach provides insights about the present moment. Further studies could benefit from samples with a stratified proportional distribution, according to the functions performed by the professional caregivers or the length of contact with the older people.

The treatment of missing data is done through elimination or imputation techniques. While elimination excludes subjects or omits cases, mean imputation is a more conservative technique. The imputation of the average, in the missing data, calculated from the available scores, is a simple method since the missing data are filled in and subsequently analysed.

### Supplementary Information

Supplementary Table 1.

## Data Availability

The datasets generated during and/or analysed during the current study are available from the corresponding author on reasonable request.
