# Supplementary material for: Fear of COVID-19 among professional caregivers of the elderly in Central Alentejo, Portugal
Source: Sci Rep. 2024 Feb 7;14:3131. doi: 10.1038/s41598-024-52993-6 (PMC10850084; doi:10.1038/s41598-024-52993-6)
Supplement: Supplementary file 1 — Supplementary Table 1. [file 41598_2024_52993_MOESM1_ESM.docx]

**Fear of COVID-19 among professional caregivers of the elderly in Central Alentejo, Portugal.**

**Suplementar Table 1 Multivariable linear regression analysis with fear against COVID-19 as the dependent variable**

| *Coefficients^a^* | | | | | | | | | | |
| --- | --- | --- | --- | --- | --- | --- | --- | --- | --- | --- |
| Model | | Unstandardized  Coefficients | | Standardized  Coefficients | t | Sig. | 95,0% Confidence Interval for B | | Collinearity Statistics | |
|  |  | B | Std. Error | Beta |  |  | Lower Bound | Upper Bound | Tolerance | VIF |
| 1 | (Constant) | 21,015 | ,251 |  | 83,772 | ,000 | 20,522 | 21,508 |  |  |
|  | EducationLevel | -2,828 | ,640 | -,172 | -4,420 | <,001 | -4,085 | -1,572 | 1,000 | 1,000 |
| 2 | (Constant) | 18,312 | ,771 |  | 23,754 | <,001 | 16,798 | 19,826 |  |  |
|  | Educational level (0-≤basic; 1-Higher Education) | -2,755 | ,634 | -,167 | -4,346 | <,001 | -3,999 | -1,510 | ,999 | 1,001 |
|  | Gender (0 male; female=1) | 2,958 | ,799 | ,143 | 3,704 | <,001 | 1,390 | 4,526 | ,999 | 1,001 |
| 3 | (Constant) | 17,985 | ,772 |  | 23,298 | <,001 | 16,469 | 19,501 |  |  |
|  | Educational level (0-≤basic; 1-Higher Education) | -2,695 | ,630 | -,164 | -4,281 | <,001 | -3,931 | -1,459 | ,998 | 1,002 |
|  | Gender (0 male; female=1) | 2,684 | ,797 | ,129 | 3,367 | <,001 | 1,119 | 4,250 | ,988 | 1,012 |
|  | Suspicious symptoms (0-16) | ,221 | ,068 | ,124 | 3,236 | ,001 | ,087 | ,355 | ,988 | 1,012 |
| 4 | (Constant) | 17,545 | ,794 |  | 22,099 | <,001 | 15,986 | 19,104 |  |  |
|  | Educational level (0-≤basic; 1-Higher Education) | -2,600 | ,629 | -,158 | -4,134 | <,001 | -3,835 | -1,365 | ,994 | 1,006 |
|  | Gender (0 male; female=1) | 2,553 | ,797 | ,123 | 3,203 | ,001 | ,988 | 4,118 | ,982 | 1,018 |
|  | Suspicious symptoms (0-16) | ,219 | ,068 | ,123 | 3,219 | ,001 | ,085 | ,353 | ,988 | 1,013 |
|  | Flu Vaccine (0-no; 1-yes) | 1,027 | ,456 | ,086 | 2,252 | ,025 | ,131 | 1,922 | ,989 | 1,011 |
| a. Dependent Variable: FCV-19S | | | | | | | | | | |
